# Timberol® Inhibits TAAR5-Mediated Responses to Trimethylamine and Influences the Olfactory Threshold in Humans

**DOI:** 10.1371/journal.pone.0144704

**Published:** 2015-12-18

**Authors:** Ivonne Wallrabenstein, Marco Singer, Johannes Panten, Hanns Hatt, Günter Gisselmann

**Affiliations:** 1 Department of Cell Physiology, Ruhr-University Bochum, Bochum, Germany; 2 Symrise AG, Holzminden, Germany; The University of Tokyo, JAPAN

## Abstract

In mice, trace amine-associated receptors (TAARs) are interspersed in the olfactory epithelium and constitute a chemosensory subsystem that is highly specific for detecting volatile amines. Humans possess six putative functional TAAR genes. Human TAAR5 (hTAAR5) is highly expressed in the olfactory mucosa and was shown to be specifically activated by trimethylamine. In this study, we were challenged to uncover an effective blocker substance for trimethylamine-induced hTAAR5 activation. To monitor blocking effects, we recombinantly expressed hTAAR5 and employed a commonly used Cre-luciferase reporter gene assay. Among all tested potential blocker substances, Timberol®, an amber-woody fragrance, is able to inhibit the trimethylamine-induced hTAAR5 activation up to 96%. Moreover, human psychophysical data showed that the presence of Timberol® increases the olfactory detection threshold for the characteristic fishy odor of trimethylamine by almost one order of magnitude. In conclusion, our results show that among tested receptors Timberol® is a specific and potent antagonist for the hTAAR5-mediated response to trimethylamine in a heterologous system. Furthermore, our data concerning the observed shift of the olfactory detection threshold *in vivo* implicate that hTAAR5 or other receptors that may be inhibited by Timberol® could be involved in the high affinity olfactory perception of trimethylamine in humans.

## Introduction

Trimethylamine (TMA) is an organic compound with a characteristic fishy odor. The tertiary amine arises by choline metabolism from precursors in food digestion and is decomposed into odorless trimethylamine oxide by the liver enzyme flavin monooxygenase (FMO3). Elevated TMA levels occurring in human urine, sweat or breath are thought to be caused by reduced FMO3 production or altered FMO3 function, resulting in fish odor syndrome or trimethylaminuria [1–3]. The FMO3 metabolic capacity is altered during menstrual periods or pregnancy, which suggests that sex hormones might play a role [4–6]. Elevated TMA levels in vaginal secretions appear after microbial degradation of trimethylamine oxide. Concerning the clinical aspects, this is the case in infectious diseases of the urinary tract or the vagina mostly caused by bacterial vaginosis [7, 8].

In addition to canonical odorant receptors (ORs), trace amine-associated receptors (TAARs) are present in the vertebrate main olfactory epithelium (OE) [9, 10]. TAARs are highly specific in detecting amine compounds [11–13]. Humans possess six putatively functional TAAR genes [14]. Using qPCR, five have been detected in the OE, with human TAAR5 (hTAAR5) at the highest level [15]. Vallender et al. showed that TAAR5 is the most conserved TAAR gene among investigated primate species and that it may have a significant functional role [16]. In heterologous systems, TAAR5 of rodents, humans and macaques can be activated by TMA [9, 11, 17, 18]. Thus, TAAR5 might be the molecular basis for the TMA detection, and it seems to be a conserved feature among different mammalian species. While mice produce gender-specific amounts of urinary TMA levels and were attracted by TMA, this odor is repellent to rats and aversive to humans [19], indicating that there must be species-specific functions. By tracing axonal projections of TAAR-expressing OSNs, it was shown that TAARs constitute an olfactory subsystem in mice [12, 20]. Furthermore, a homozygous knockout of murine TAAR5 abolished the attraction behavior to TMA [19]. Thus, it is concluded that TAAR5 itself is sufficient to mediate a behavioral response at least in mice.

The question remains to what extent TAAR5 is involved in the perception of TMA in humans. To provide more clarity, we endeavored to find an antagonist for hTAAR5-mediated responses to TMA in a heterologous system. Subsequently, we tested the antagonist on human perception in a psychophysical assay. In the present study, we were able to identify Timberol® as a potent blocker substance *in vitro* and showed that Timberol® also influences the sensitivity of humans toward TMA.

## Materials and Methods

### Cre-luciferase assay

We adapted the optimized protocol of Zhuang and Matsunami for measuring receptor activity with the Dual-Glo Luciferase Assay System (Promega) [21]. HANA3A cells were maintained under standard conditions in DMEM supplemented with 10% FBS and 100 units/ml penicillin and streptomycin at 37°C. Cells (approximately 15,000 cells/well) were plated on poly-D-lysine–coated 96-well plates (NUNC) and transfected after 24 h with the *FuGENE®* HD (Promega) transfection reagent according to the manufacturer’s protocol. In a 96-well plate, we placed 18 μl transfection reagent, 1–5 μg receptor plasmid, 2 μg pGL4-luciferase reporter, 1 μg pRL-TK-*Renilla* reporter, 0.5 μg G-protein α_olf_, 1 μg receptor transport protein (RTP1S) and 1 μg M3 muscarinic acetylcholine receptor (for hOR51E1) to ensure cell surface expression [22, 23]. Cells were stimulated 24 h after transfection for 4 h at 37°C with test substances diluted in CD 293 medium (1x) (Life Technologies) with 2 mM L-glutamine (Life Technologies) added. After stimulation with test substances, recombinant hTAAR5 activation elevated cAMP and subsequently induced Cre-dependent expression of the reporter gene luciferase [18, 24]. Expression rates of Cre-luciferase were monitored by luminescent enzymatic reactivity. Thus, luminescence signals correlate with receptor activation. The *Renilla* luciferase reporter driven by a constitutively active TK-promoter (pRL-TK-*Renilla*) served as an internal control to determine cell viability and transfection efficiency. We normalized firefly luciferase activity to the *Renilla* luciferase signal for all wells. Stimulation with test substances in each independent experiment (n) was performed in duplicates and luciferase activity was calculated as mean. Mock-transfected cells were stimulated to exclude unspecific responses to the tested substances.

### Chemicals

All tested substances were provided by Symrise AG in Holzminden or purchased from Sigma-Aldrich. Substances were dissolved in DMSO or Ringer to prepare 100 mM stock solutions.

### Participants

Subjects were recruited among the R&D department of Symrise AG. Experiments were approved by the ethics committee of Symrise AG. The panel consisted of 27 individuals (18 females; 9 males) between 19 and 55 years of age (mean: 33.8 years; standard deviation: 10.9) and was not particularly trained in sensory evaluations. Panelists were instructed to refrain from smoking, eating spicy food and drinking coffee prior to the sensory evaluations.

### Olfactory threshold measurements

Olfactory detection thresholds were assessed in aqueous solutions using a best estimate threshold approach as described in ISO 13301. In brief, samples containing different concentrations of analyte were presented in ascending order in a 3-alternative forced-choice design. Each concentration set consisted of three samples with two samples containing only solvent and one deviating sample containing a defined amount of trimethylamine (TMA) or triethylamine (TEA). Samples were presented in amber vials (30 ml) coded with three digits and filled with 10 ml of solution, and panelists were required to designate the sample that differed from the others.

Thresholds of TMA and TEA were measured in buffered aqueous solution i) without additive ii) with 1% (m/m) Timberol® and in the case of TMA iii) with 0.1% Mugetanol (isointense to 1% Timberol®) and iv) 0.01% Ambrocenide® (isointense to 1% Timberol®). These experiments were performed separately on different days with the same panelists. In the case of testing in the presence of fragrance components (Timberol®, Mugetanol or Ambrocenide®), all three samples of a given concentration set contained the same amount of fragrance components and differed only in the presence/absence of amine (TMA or TEA). In each session, a total of 10 concentration sets ranging from 1.95×10^−8^ to 1.00×10^−5^% (m/m) TMA were evaluated. In the case of of TEA the range was between 9.77×10^−5^ and 5.00×10^−2^% (m/m). The concentration doubled in each step.

The aqueous solutions were buffered with TRIS (50 mM), and the pH was adjusted to 9 to have approximately 13% of the TMA, respectively 17% of the TEA, in a deprotonated and thus volatile form. Furthermore, an odorless emulgator (solubilisant) was added to a final concentration of 3% (m/m) to dissolve Timberol® and the other lipophilic fragrance components.

### Statistics

For statistical analysis of Cre-luciferase assay data, we used two-tailed unpaired Student`s t-test. Given error bars represent SEM. Curve fitting was performed with the Hill equation using SigmaPlot V8.0 (Systat Software, San Jose, CA). Sensory data was analysed using non-parametric statistical tests. The overall difference between the group thresholds was determined by a Kruskal-Wallis test, followed by a Dunn post-hoc test for the respective pairwise comparisons.

## Results

### Cre-luciferase assay

#### Blocker Screening

**Fig 1:**
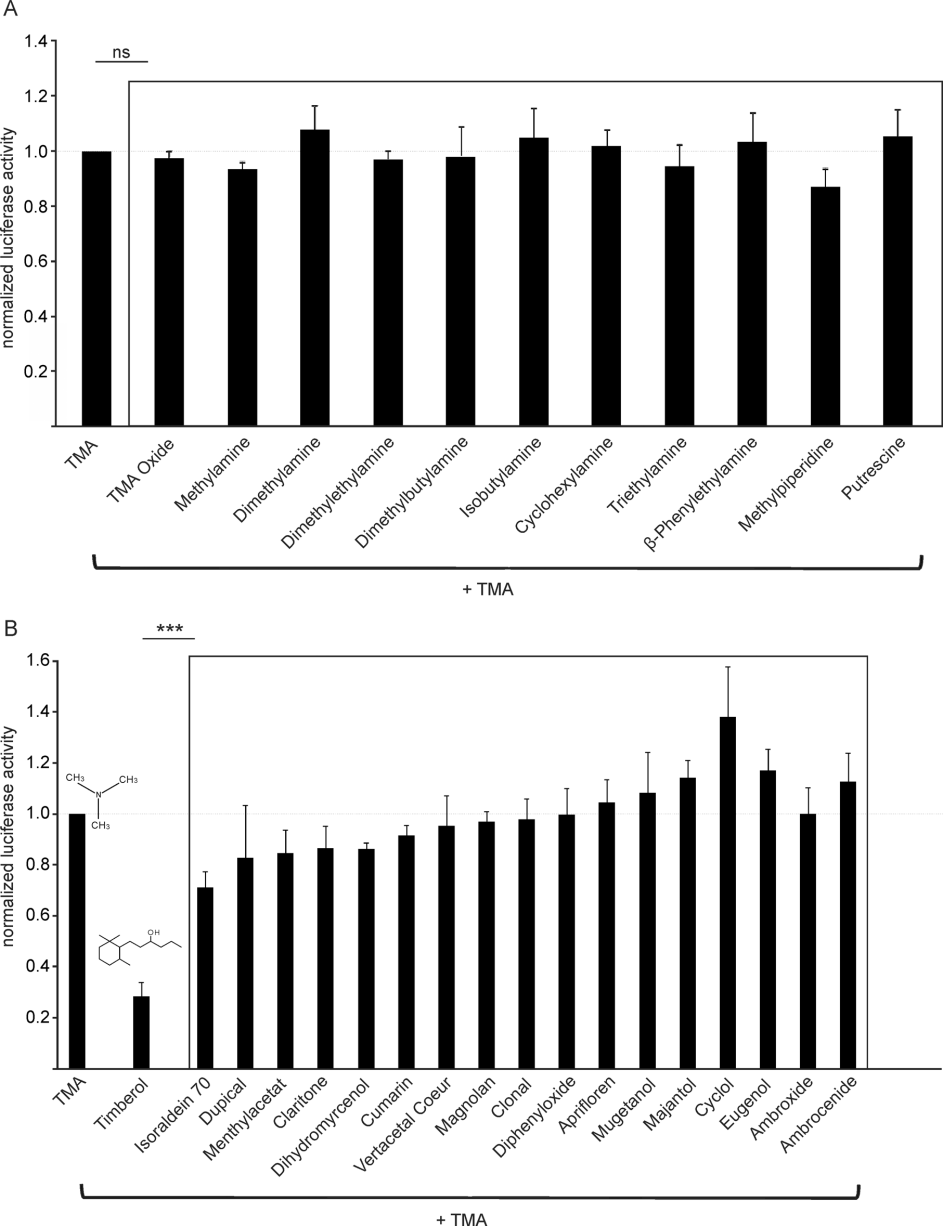
Blocker Screening. Each of the applied mixtures contained the agonist TMA (300 μM) and a putative antagonistic substance (100 μM). Responses were normalized to the agonist TMA alone. (A) No significant blocking effects of structural TMA analogs (n = 3–6). (B) Significantly stronger blocking effect of Timberol® compared to the other tested substances (n = 3–7). *** p<0.001

To identify blocker substances for hTAAR5-mediated responses to TMA, we used the Cre-luciferase reporter gene assay. Human TAAR5-transfected HANA3A cells were stimulated with a mix of the agonist TMA (300 μM) and 100 μM of a potential blocker substance (Fig 1). An initial screening was performed with 11 chemically related structures of the hTAAR5 agonist TMA in which none of the chemical TMA analogs showed a significant reduction of hTAAR5-mediated TMA responses (Fig 1A). Subsequent screening with 18 non-related chemical structures revealed that 100 μM Timberol® was able to inhibit hTAAR5 responses to 300 μM TMA by 72% ± 6%. This blocking effect was significantly stronger (p<0.001) than that observed for all other tested substances (Fig 1B).

**Fig 2:**
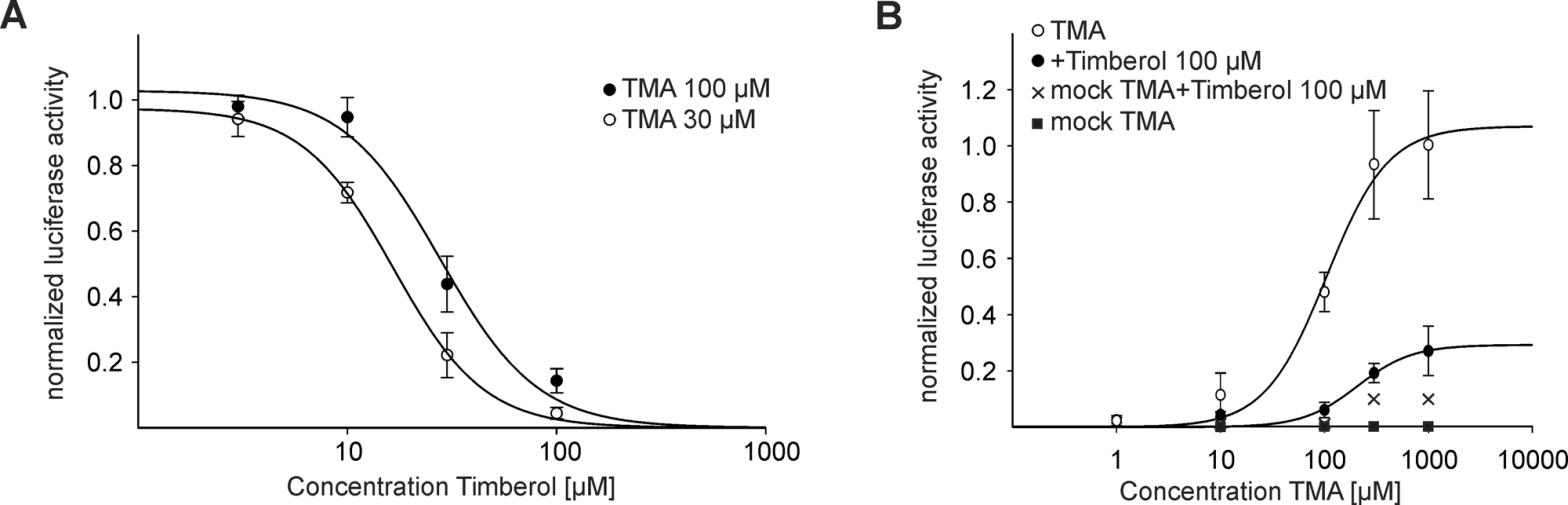
Blocker properties of Timberol®. (A) Concentration-inhibition curve (n = 3). Each response was normalized to the agonist TMA alone. Calculated IC_50_ for 100 μM TMA on hTAAR5 was 27.8 μM ± 6.5 μM, and the IC_50_ for 30 μM TMA was 16.6 μM ± 0.9 μM. The curve shift was significant (* p<0.05). (B) Concentration-response curve (n = 3). Each response was normalized to forskolin. Calculated EC_50_ for TMA on hTAAR5 was 104.8 μM ± 26.02 μM. TMA in the presence of Timberol® (100 μM) revealed an EC_50_ of 208.4 μM ± 95.10 μM. Curve shift was significant (* p<0.05).

For more detailed information concerning the type of antagonism (competitive vs. non-competitive) in the following experiments, we characterized the pharmacological profile of Timberol®. To test the concentration dependency of the Timberol® block, we measured a concentration-inhibition curve (Fig 2A). The blocking effect was increased with increasing concentrations of Timberol® and decreased with increasing concentrations of the agonist TMA. The latter was reflected by a significant concentration-inhibition curve shift (Fig 2A). The half maximal inhibitory concentration (IC_50_) of Timberol® was calculated with 27.8 μM (TMA 100 μM) and 16.6 μM (TMA 30 μM) (Fig 2). Thus, a Timberol® concentration of 30 μM was sufficient to block average hTAAR5 responses at least 50%, ~56% of the response to 100 μM TMA, and ~78% of the response to 30 μM TMA. A Timberol® concentration of 100 μM was able to almost completely block the response to 30 μM TMA (blocking effect 96% ± 1.7%) as well as 100 μM TMA (blocking effect 86% ± 3.6%) (Fig 2A). Further, we measured concentration-response relationships for TMA alone and TMA in a mix with Timberol®. We revealed a significant shift of calculated EC_50_ values from 104.8 μM ± 26.02 to 208.4 μM ± 95.10 μM (Fig 2B). The hTAAR5 activity induced by the saturating TMA concentration of 1 mM was strongly reduced in the presence of Timberol® (blocking effect 73 ± 8.7%).

#### Timberol® blocker specificities

**Fig 3:**
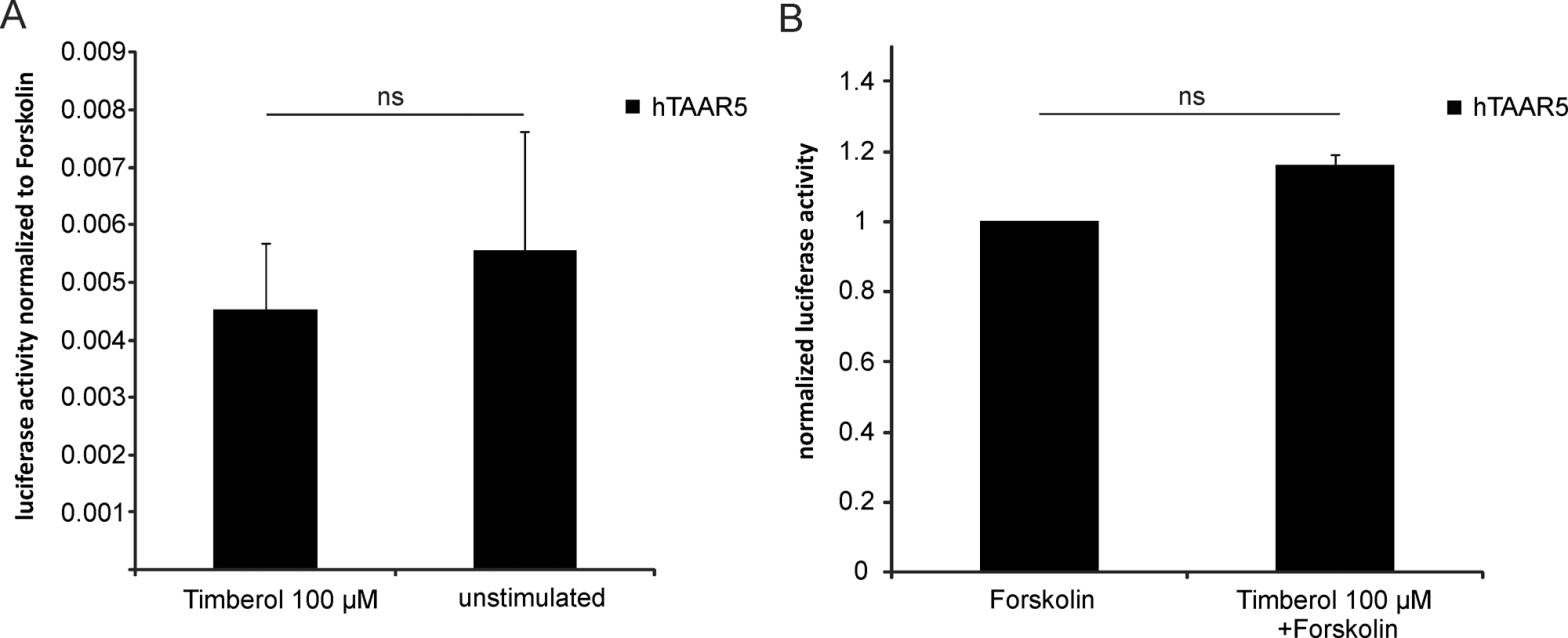
Blocking effect of Timberol® is mediated by hTAAR5. Timberol® (100 μM) was either applied alone (normalized to unstimulated control) or in a mixture with 10 μM forskolin (normalized to forskolin alone) (n = 3–6). Timberol® did not affect measured luciferase activity (p>0.05).

To determine whether the observed blocking effect of Timberol® is actually mediated by the receptor hTAAR5, we performed two different control experiments (Fig 3). Both controls aimed to exclude the possibility that Timberol® independent of hTAAR5 modulates the level of intracellular cAMP. To determine whether Timberol® affects the basal intracellular cAMP level, we tested hTAAR5-transfected cells stimulated with Timberol® alone. Even if the basal intracellular cAMP level of unstimulated controls is very low (< 1% of maximum response by forskolin), we could not observe any significant reduction of luciferase activity by Timberol® (Fig 3). To determine whether Timberol® reduces cAMP levels produced by adenylyl cyclase III after receptor activation, we stimulated hTAAR5-transfected HANA3A cells with forskolin, an adenylyl cyclase III activator. Timberol® mixed with forskolin compared to forskolin alone revealed about 16% higher cAMP levels, although this observed difference in measured luciferase activities is not significant as well (Fig 3).

**Fig 4:**
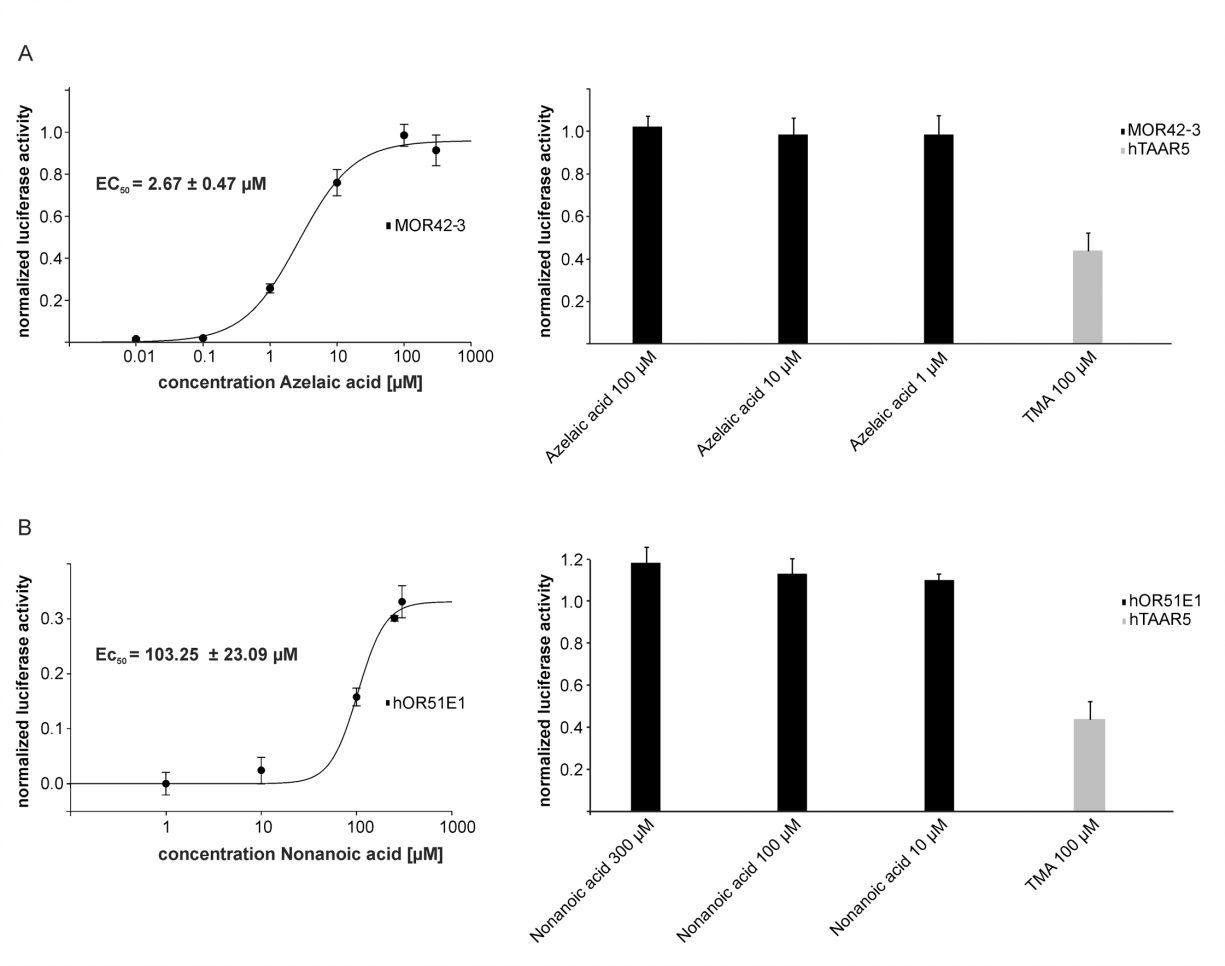
Blocking effect of Timberol® is exclusively mediated by hTAAR5. Each response was normalized to corresponding agonist alone. Left: Concentration-response curves (n = 3) of (A) azelaic acid and MOR42-3 and (B) nonanoic acid and hOR51E1. Right: Blocking effects (n = 3) of Timberol® (30 μM) on (A) MOR42-3-mediated responses to azelaic acid and (B) hOR51E-mediated responses to nonanoic acid. No significant blocking effects were observed. Gray bars represent the blocking effect of Timberol® (30 μM) on hTAAR5-mediated responses to TMA in a concentration close to calculated EC_50_.

We further sought to determine whether the Timberol® block could be exclusively mediated by hTAAR5. To do so, we picked a murine (MOR42-3) and a human olfactory receptor (hOR51E1) with known ligands. First, we constructed concentration response curves for both receptors to determine the EC_50_ values of agonistic action for each (Fig 4A and 4B; left part). Afterward, we tested the impact of Timberol® on MOR42-3- and hOR51E1-mediated responses to the respective agonists with concentrations below and above the calculated EC_50_ values. In addition to the blocker properties of Timberol® concerning hTAAR5 and TMA, we could not observe any inhibitory impact on other tested ORs (Fig 4A and 4B; right part).

In turn, we observed that Timberol® is able to block not only hTAAR5-mediated responses to TMA but also murine TAAR5 responses (see S1 Fig), which strongly supports that antagonistic effects are mediated by TAAR5. As for the hTAAR5 shown, the blocking effect of Timberol® on murine TAAR5 also depends on the agonistic TMA concentration. The observed blocking effect at the previously reported EC_50_ value of 1 μM TMA [18] is 34% ± 2% weaker than that for hTAAR5. The smaller impact of Timberol® can probably be explained by differences in the protein sequence of TAAR5, which might also be responsible for species-specific differences in the agonistic profiles and receptor sensitivities [13, 14, 18].

Experiments utilizing heterologous receptor expression revealed that Timberol® is able to nearly completely block hTAAR5-mediated responses to TMA.

### Human olfactory psychophysics

In a second step, the influence of Timberol® on the perception of TMA was investigated using a psychophysical approach. Therefore we investigated the detection thresholds of TMA alone and in the presence of suprathreshold concentrations of Timberol®. Threshold measurements were performed in a 3-alternative forced-choice design according to ISO 13301. A total of 10 concentrations (binary dilution steps) ranging from 1.95×10^−8^ to 1.00×10^−5^% (m/m) TMA were examined.

**Fig 5:**
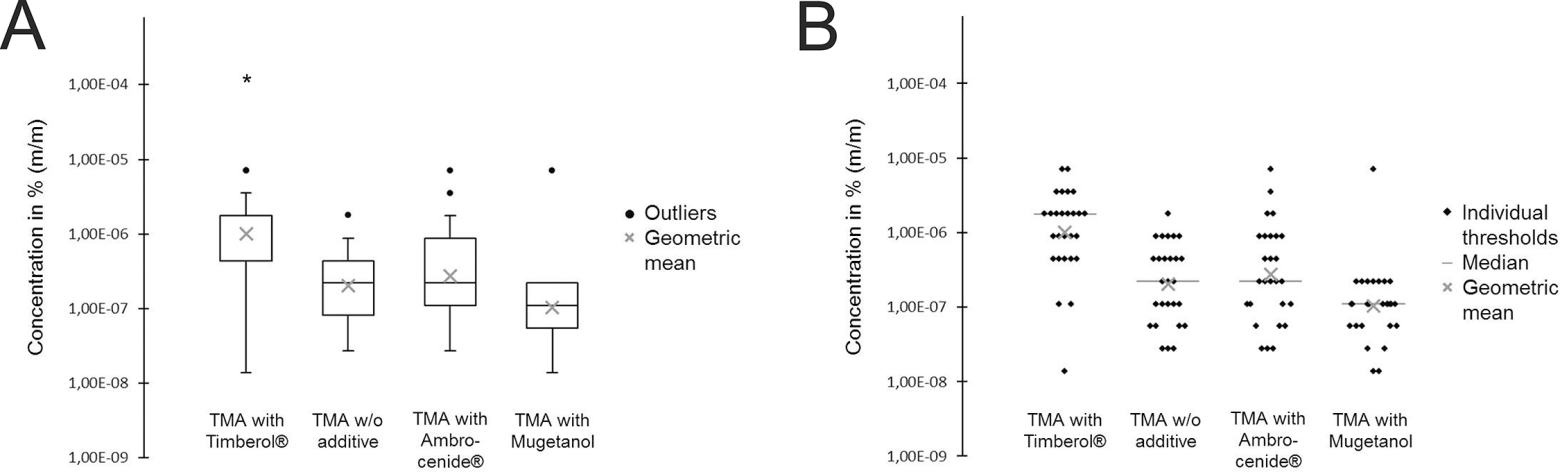
(A) Box plots of TMA thresholds. In the presence of Timberol®, the threshold of TMA was shifted to a higher concentration compared to TMA alone or in the presence of Ambrocenide® and Mugetanol.* p<0.05. (B) Scatterplots of individual thresholds with TMA.

**Fig 6:**
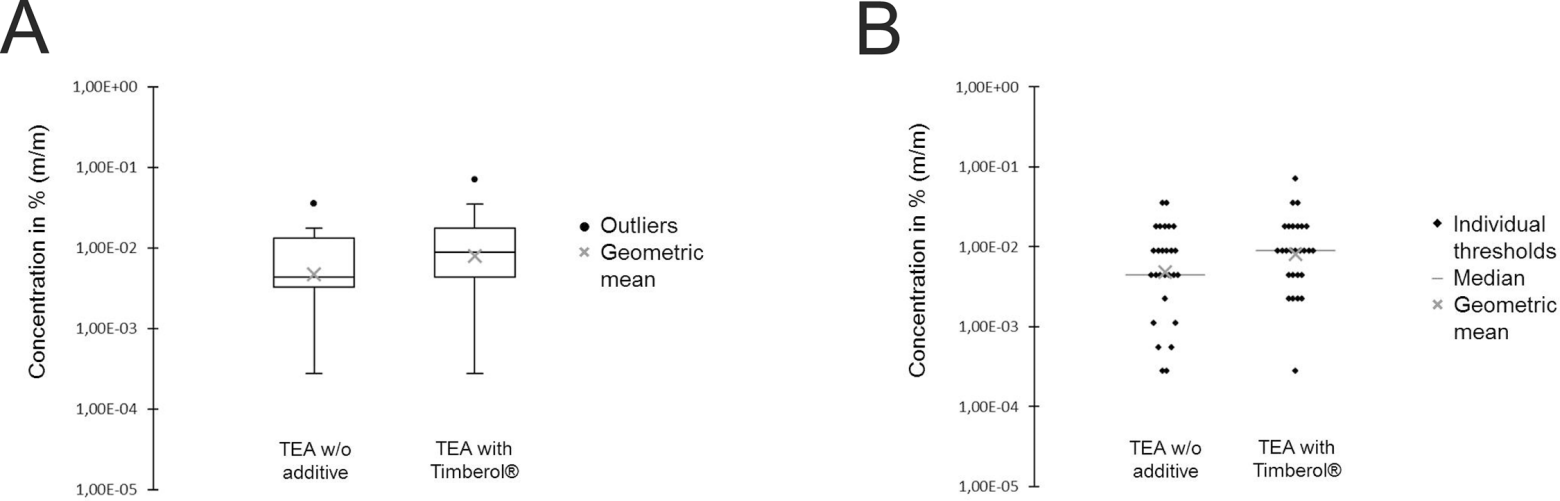
(A) Box plots of triethylamine (TEA) thresholds. The addition of Timberol® does not significantly alter the TEA threshold. (B) Scatterplots of individual thresholds with TEA.

In the presence of Timberol®, the group detection threshold was shifted to a significantly higher concentration compared to samples without additive or in the presence of Ambrocenide® and Mugetanol which does not inhibit hTAAR5 (Fig 5). This indicates that a block of hTAAR5 reduces the sensitivity of TMA. Moreover, the threshold of triethylamine (TEA), which is not an agonist of hTAAR5, was measured in presence and absence of Timberol® in analogy to TMA. In contrast to TMA the threshold of TEA (0.005% (m/m)) was not significantly shifted in the presence of Timberol® (0.008% (m/m)) (Fig 6).

## Discussion

The ability of TAAR5 to detect TMA is sustained within mammals from rodents to higher primates. Human TAAR5 as well as TAAR5 of phylogenetically closely related macaques are specifically activated by TMA [17, 18]. In addition, with less efficacy and potency, hTAAR5 can be activated by dimethylethylamine (DMEA). Remarkably, among amine-based compounds, these two hTAAR5 agonists do have by far the lowest detection thresholds in humans (DMEA 0.0076 ppm and TMA 0.00047 ppm compared to methylamine 180 ppm, dimethylamine 34 ppm or triethylamine 2.2 ppm) [25]. People were up to nearly 4x10^5^ less sensitive in detecting TMA analogs (refers to methylamine versus TMA). The human olfactory system obviously has an extraordinarily high affinity to TMA. This fits quite well with the generally recognized feature of TAARs as ultrasensitive amine detectors, at least in mice [11, 12]. Now the question remains whether hTAAR5 mediates the olfactory perception of such low TMA concentrations in humans. To answer this question, we struggled to identify a blocker of hTAAR5 in order to perform sensory studies with humans. Our experiments clearly showed that Timberol® is able to almost completely inhibit (~96%) hTAAR5-mediated responses to TMA in a heterologous system. As is typical for competitive antagonists, blocker effectiveness is increased with antagonistic Timberol® concentration and depends on the amount of agonistic TMA. There was a significant EC_50_ shift in the presence of Timberol®. Thus, Timberol® lowers hTAAR5 sensitivity. Furthermore, although the blocking effect decreases with increasing amounts of agonistic TMA, Timberol® is still able to inhibit hTAAR5-mediated responses to a saturating TMA concentration by approximately 73%. These observed characteristics strongly support an involvement of a non-competitive antagonistic effect. It was previously shown that the chemical structure of competitive antagonists is often similar to the agonist [26–28]. Because Timberol® and TMA completely differ in their chemical structure, the use of a different binding site typical for an allosteric modulator is likely. Psychophysical data of TMA perception showed that the olfactory detection threshold of TMA is shifted by almost one order of magnitude from 2.0×10^−7^% (m/m) to 1.0×10^−6^% (m/m) in the presence of Timberol®. An isointense concentration of Mugetanol on the other hand does not significantly influence the TMA perception (Fig 5). Our determined TMA detection threshold is in accordance with the normal TMA threshold published in standard literature [25]. Timberol® abolished the ability to perceive TMA around perithreshold concentrations whereas the threshold of triethylamine which is not an agonist of hTAAR5 was not significantly altered (Fig 6). We speculate that hTAAR5, a previously described high-sensitivity amine detector in mice, might be involved in the olfactory perception of TMA in humans as well. However, the observed blocking effect *in vivo* could also be caused by centrally based effects. Further, it is probable, that also olfactory receptors are activated by TMA. That additional ORs play a role in sensing higher TMA concentrations was implicated by a previous study, were people with a specific TMA anosmia have ~10^3^ higher olfactory detection thresholds [25]. This means that TMA anosmics cannot smell low but higher TMA concentrations. Here, additional ORs are involved or genetic variations of the hTAAR5 gene might be responsible for reduced receptor affinity to TMA; as such a correlation was previously described for ORs [29–31]. However, TMA anosmics did not show any examined genetic polymorphism (*single nucleotide polymorphisms*, SNPs) within the open reading frame of their hTAAR5 genes [18]. Supported by the results of the present study, this implies that the molecular reason for the observed TMA anosmia is not caused by a SNP within the open reading frame of hTAAR5 but possibly by a mutation elsewhere in the hTAAR5 gene or in a gene regulatory element. Moreover, reduced perception may be independent of the odorant binding properties of the receptor but also caused by reduced receptor expression rates or alterations in higher brain processing. Another argument for considering different TMA detectors is that it is commonly believed that odors were coded in a redundant fashion. Malnic et al. stated that an increase in the concentration of an odorant leads to the recruitment of additional ORs and a consequent change in the receptor code [32]. Therefore, differential receptor output patterns potentially explain differences in perceived odor qualities of the same chemical structure as well [32]. While hTAAR5 seems to be involved in the fishy odor perception at lower TMA concentrations, additional low affinity ORs might consequently detect higher TMA concentrations with a more ammonia-like odor quality. In the end, that TMA detection is executed by additional ORs cannot be excluded. In turn, many ORs were reported to be activated by β-ionone, but only OR5A1 seems to be necessary for the highly sensitive odor perception of β-ionone [33, 34].

Axonal projections of TAAR expressing OSNs in humans have not yet been examined, and the functional relevance of TMA sensing in humans also remains unclear. In contrast to mice, TMA excretion via urine is not gender-specific [6] but may be altered by female sex hormones [4–6]. On the other hand, elevated TMA excretion via urine or vaginal secretions occurs in pathophysiological situations as well and the fishy body odor can be a product of disease [7]. TMA is further generated by bacteria during spoilage and is responsible for the malodor of rotten fish [3, 35]. Horowitz et al. showed that hTAAR5 can be activated by rotten fish and suggested a possible role in providing sensory input about food harboring pathogenic microorganisms [17]. Whether the TAAR5 activation by TMA elicits specific behavioral output like avoidance behavior in humans still needs to be examined.

## Conclusion

We identified Timberol® as an antagonist for TAAR5-mediated responses to TMA in a heterologous system. Moreover, we showed that Timberol® reduces the sensitivity to the fishy odor of TMA in a psychophysical approach. We speculate that hTAAR5 or other ORs blocked by Timberol are good candidates for receptors involved in the perception of TMA. However, the true correlation between olfactory perception *in vivo* and determined receptor properties in *in vitro* assays stays a major challenge in olfactory science.

## Supporting Information

S1 Fig Timberol® inhibits mTAAR5-mediated responses to TMA. Responses were normalized to agonist alone. The concentration of Timberol® was 100 μM. Error bars represent SEM. (n = 3). ** p≤0.01.
(TIF)
