# Supplementary figures and images for: Timberol® Inhibits TAAR5-Mediated Responses to Trimethylamine and Influences the Olfactory Threshold in Humans
Source: PLoS One. 2015 Dec 18;10(12):e0144704. doi: 10.1371/journal.pone.0144704 (PMC4684214; doi:10.1371/journal.pone.0144704)

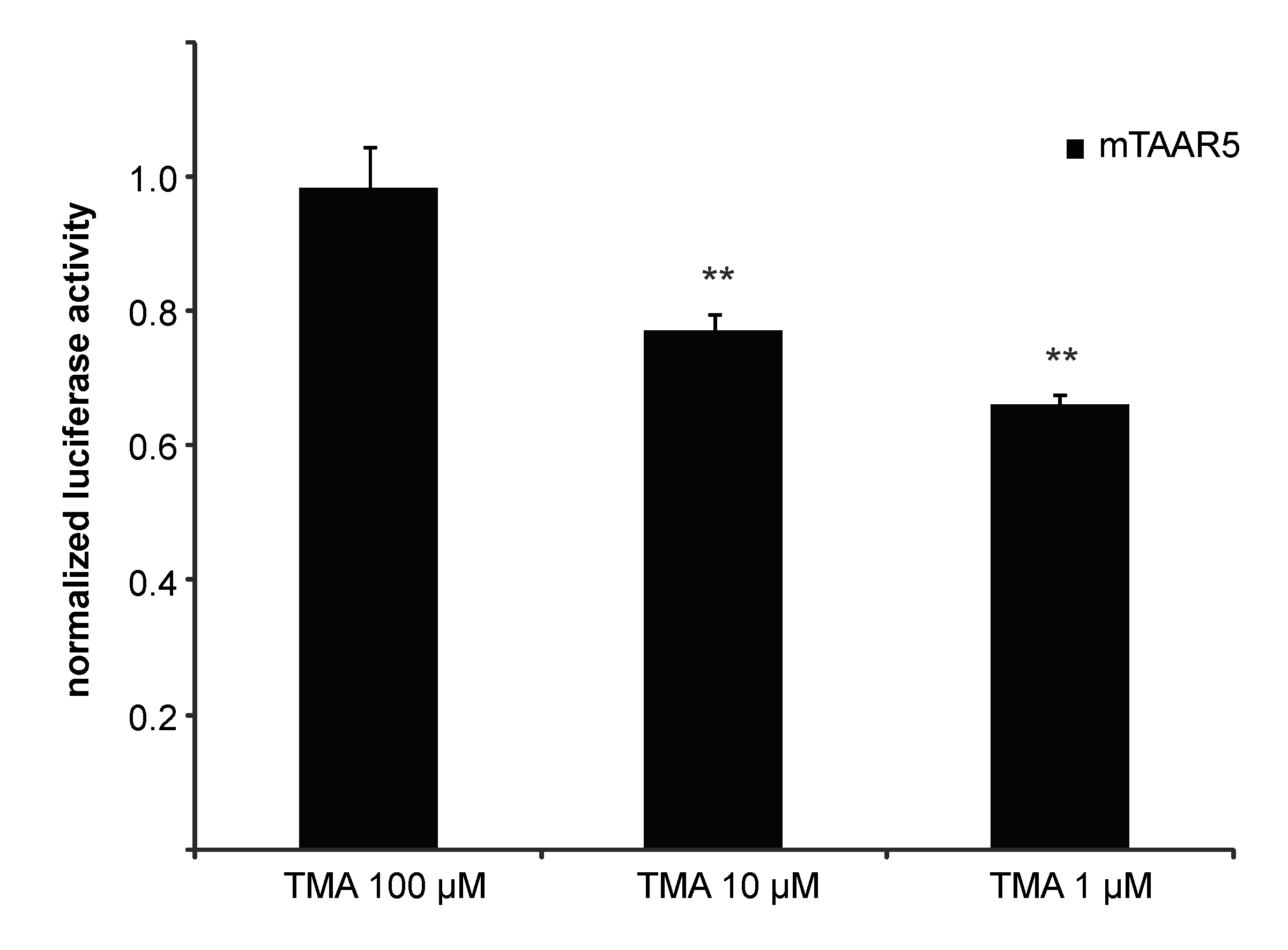

Supplement: S1 Fig — Responses were normalized to agonist alone. The concentration of Timberol® was 100 μM. Error bars represent SEM. (n = 3). ** p≤0.01. (TIF) [file pone.0144704.s001.tif]
